# Net Primary Productivity and Edaphic Fertility in Two Pluvial Tropical Forests in the Chocó Biogeographical Region of Colombia

**DOI:** 10.1371/journal.pone.0168211

**Published:** 2017-01-23

**Authors:** Harley Quinto-Mosquera, Flavio Moreno

**Affiliations:** 1 Programa de Biología, Facultad de Ciencias Básicas, Universidad Tecnológica del Chocó “Diego Luis Córdoba”, Quibdó, Colombia; 2 Departamento de Ciencias Forestales, Facultad de Ciencias Agrarias, Universidad Nacional de Colombia Sede Medellín, Medellín, Colombia; Tennessee State University, UNITED STATES

## Abstract

The net primary productivity (NPP) of tropical forests is a key process of the carbon cycle and therefore for the mitigation of global climate change. It has been proposed that NPP is limited by the availability of soil nutrients in lowland tropical forests and that belowground NPP decreases as edaphic fertility increases. This hypothesis was evaluated in two localities (Opogodó and Pacurita) of the Chocó Biogeographical region, one of the rainiest of the world, where the aboveground (litter and wood) and belowground (fine and coarse roots) components of NPP were measured. Fertility parameters (pH, nutrients, and texture) were also determined and related to NPP. Total NPP was similar between locations (23.7 *vs*. 24.2 t ha^-1^ year^-1^ for Opogodó and Pacurita, respectively). However, components of NPP showed differences: in Pacurita, with steeper topography, NPP of wood and coarse roots were higher; therefore, differences of topography and drainage between localities probably affected the NPP of wood. On the other hand, soils of Opogodó, where NPP of fine roots was higher, showed higher contents of sand, N+, and organic matter (OM). With the increase of pH, OM, N+, K, Mg, and sand, the NPP of leaves and fine roots as well as the percentage of NPP _belowground_ also increased, which suggests NPP limitation by multiple nutrients. The increase of NPP _belowground_ with the availability of edaphic nutrients evidenced a redistribution of the aboveground and belowground components of NPP with the increase of soil fertility in oligotrophic systems, probably as a mechanism to improve the capture of resources.

## Introduction

Tropical forests comprise the terrestrial ecosystems with the highest net primary productivity (NPP) on the planet [1, 2] and account close to 30% of total NPP [1, 3]. For this reason, they are considered fundamental for the carbon balance and the mitigation of global climate change [4, 5]. Several studies have documented that NPP is determined by environmental factors such as temperature, precipitation, sunlight, soil properties, and CO_2_ concentration in the air, among others [6, 7, 8, 9]. Nevertheless, our understanding on how these factors affect the NPP is still very limited.

For several decades, the role of soil nutrients as limiting factors for NPP in tropical forests has garnered attention; furthermore, the hypothesis that, in low-altitude tropical forests, the NPP is principally limited by the edaphic availability of P has been put forth [10, 11, 12]. However, few studies have found significant correlations between NPP and the edaphic availability of nutrients in tropical rainforests and confirmed a positive relationship between NPP and P availability [9, 13, 14].

The relationship between the content of soil nutrients and the NPP in tropical forests varies in accordance with the NPP component evaluated; it is postulated that, when nutrients increase, a change occurs in the aboveground and belowground allocation of NPP. As a result, two hypotheses have been proposed about the effect of soil resources on NPP [15]. The first, called the “differential allocation hypothesis” puts forth that a higher availability of edaphic resources increases the total NPP, with a proportional increase in the aboveground NPP (wood and foliage) and reduction in the belowground NPP (roots). The second hypothesis, called the “constant allocation hypothesis” proposes that the higher soil fertility increases total NPP and that the amounts allocated to aboveground and belowground components remain relatively constant.

Studies on NPP and its relationship with edaphic fertility in lowland tropical forests have covered a limited range of environmental conditions, specifically they have only evaluated ecosystems with rainfall under 5,000 mm annually [7, 9]. Nevertheless, the strong influence of precipitation on the functioning of ecosystems is well known [7, 16]; for example, it has been documented that excessive rainfall in the tropics results in nutrient losses due to leaching and run-off [17] and affects weathering processes, abundance and type of clays, cation exchange capacity (CEC), saturation of bases, pH, concentration of Al, and activity of soil organisms [18]; in addition, it generates stress conditions due to anoxia, limits the decomposition of organic matter (OM), and reduces the gas exchange of roots [7, 19] and rates of mineralization and nitrification of N [16].

According to the above, it is expected that the increase of precipitation would result in a reduction in the NPP of tropical forests [7]; however, the few published studies did not evidence such reduction [9]. This study evaluates the magnitude of NPP and its components in two tropical pluvial forests in the Chocó biogeographical area, where the annual precipitation exceeds 10,000 mm [20]. Furthermore, it evaluates the influence of edaphic conditions on NPP in these ecosystems and how much the aboveground and belowground NPP are influenced by soil fertility.

## Methods

### Study area

The present study was carried out in the tropical pluvial forests of the localities of Pacuríta (municipality of Quibdó) and Opogodó (municipality of Condoto) in the department of Chocó, Colombia (Table 1). These two localities are part of the Central North ecogeographical subregion of the Chocó biogeographical region, which encompasses the high watersheds of the Atrato and San Juan rivers [20]. They are found in the geomorphological unit of Tertiary sedimentary hills, which have low altitudes and contain sandy claystone, sandstone, and limestone [21, 22].

**Table 1:** Environmental characteristics of the study sites in the tropical rainforests of Chocó, Colombia.

Variables	Opogodó	Pacurita
Municipality	Condoto	Quibdó
Latitude	5°04´079 N	5°41' 55.8” N
Longitude	76°64´74" W	76°35'59.4” W
Temperature (°C)	26–30	26
Annual rainfall (mm)	8000	10000
Altitude (masl)	70	106–130
Relative humidity (%)	90	87
Soil type (USDA)	*Typic Tropudults*–Ultisol	*Typic Tropudults*–Ultisol
Soil type (FAO)	*Haplic Acrisols*–Acrisoles	*Haplic Acrisols*–Acrisoles
Topography	Flat to slightly inclined	Slightly inclined to steep
Drainage	Imperfect to excessive	Imperfect to excessive
Geomorphologic unit	Colluvial alluvial piedmont	Structural to erosional knoll
Parent material	Sedimentary tertiary rock	Sedimentary tertiary roc
Life zone	Tropical rain forest	Tropical rain forest
Dominant tree species	*Wettinia quinaria*, *Mabea occidentalis*, *Calophyllum auratum*, *Eschweilera sclerophylla*, *Oenocarpus bataua*	*Calophyllum auratum*, *Eschweilera sclerophylla*, *Jessenia bataua*, *Protium apiculatum*, *Brosimum utile*
Dominant botany families	*Arecaceae*, *Fabaceae*, *Lecythidaceae*, *Hypericaceae*, *Sapotaceae*, *uphorbiaceae*	*Arecaceae*, *SapotaceaeLecythidaceae*, *Clusiaceae*, *Moráceae*, *Chrysobalanaceae*
Density (individuals ha^-1^)	625.6	658
Basal area (m^2^ ha^-1^)	19.12	23.15
Aerial biomass (t ha^-1^)	156.85	217.85
Fine root biomass (t h^-1^)	5.91	6.28
Fine root turnover (years^-1^)	1.17	0.62
Litter turnover (years^-1^)	3.55	3.42

Information taken from local studies [21, 23, 24, 25].

In Opogodó, the sampling was carried out in 3 one-hectare permanent plots of primary forest located in the fields of the Universidad Tecnológica del Chocó “Diego Luis Córdoba”. In Pacurita, this study was conducted in two one-hectare permanent plots in a forest reserve under the care of the same university located 6.5 Km from the municipality of Quibdó, on the road between Quibdó and Pacurita. The permission to do this study in both locations was issued by this university.

### Conceptual model

The NPP was determined from the conceptual models 1, 2, 3, and 4 [5]:

$$NPP={NPP}_{aboveground}+{NPP}_{belowground}$$ (1)

$${NPP}_{aboveground}={NPP}_{wood}+{NPP}_{litter}$$ (2)

$${NPP}_{wood}={\Delta AB}_{surviving}+{\Delta AB}_{recruited}$$ (3)

$${NPP}_{belowground}={\Delta BB}_{surviving}+{\Delta BB}_{recruited}+{NPP}_{fine\;roots}$$ (4)

Where NPP _aboveground_ is the aboveground NPP and NPP _belowground_ is the belowground NPP. In Eq 3, ΔAB _surviving_ is the increase in the aboveground biomass (AB) of trees determined as the final AB (second measurement) minus the initial AB (first measurement) of each surviving individual; ΔAB _recruited_ is the increase in the AB of recruited trees (individuals that grew to 10 cm of DBH or more in the period) calculated as the AB of new trees recorded in the second measurement minus the AB of individuals of 10cm DBH; and NPP _litter_ is the litter production. In Eq 4, BB is the biomass of the coarse roots and was estimated as 21% of AB [15]; its increase was determined similarly to the estimation of the increase of AB with Eq 2. The NPP _fine roots_ is the production of fine roots [5, 26].

### Establishment and census of plots

The five permanent plots were established in 2013; the dimension of each plot was 100 m x 100 m, divided into 25 20 m x 20 m subplots (400 m^2^); each subplot was further divided into 4 10 m x 10 m sampling units, in which soil samples were taken and the NPP was measured.

All trees with diameter at breast height (DBH) ≥ 10 cm inside the plots were inventoried. In each census, living, dead, and recruited individuals were recorded. Censuses were done in August 2013 and 2014.

### Measurement of tree diameters and heights

The circumference at breast height (at 1.30 m above the ground) in cm was measured with metric tape on all trees with DBH ≥ 10 cm in each sampling unit; afterwards, circumference values were transformed to diameter (DBH). The perimeter of DBH measurement in the tree bole was marked with yellow spray paint to guarantee that subsequent measurements are taken in the same place as the first one; both measurements were taken in areas free of nodes and branches. Additionally, all of the measured trees were marked with aluminum tags and growth habits were classified in the categories of tree, vine, liana, and palm; the vegetative characteristics and particular observations to each individual were also recorded. The heights of 40% of the trees were measured with Suunto^TM^ clinometer at fixed distances of 15 and 20 m; for the remaining 60%, heights were estimated based on the ecological group of the species with Eqs 5 and 6 [27].

Totalheightclimaxic(m)=6.28+0.607*DBH (5)

Totalheightpioneer(m)=7.42+0.41*DBH (6)

### Botanical identification

Trees were identified to the maximum possible taxonomic level (NN, species, genus, family) using the key of [28] in the herbarium of the Universidad Tecnológica del Chocó “D.L.C.” “Herbario Chocó”.

### Estimation of wood density

The values published in two international wood density databases were used to estimate this variable: one was generated in the forests of the Amazon [29] and the other in tropical forests of several regions around the world [30]; when a species or genus found in the plots was not reported in the databases, the average of the genus or family of the species was used; for individuals taxonomically indeterminate, the average density of the plot was used.

### Estimation of the aboveground (AB) and belowground biomass (BB) of trees

Since there is no local AB model, seven models created with data from very wet tropical forests of diverse areas of the world were evaluated for estimating the AB [29, 30, 31, 32]. The model developed for trees with a DBH ≥ 10 cm in wet neotropical forests [32] was selected (Eq 7) because it presented a higher mean coefficient of correlation with the AB estimated from the other models, according to a previous evaluation [27]; in addition, this model was chosen because it includes the most important AB predictor variables, such as DBH, total height, and wood density, and therefore the estimations obtained better represented the natural variability of AB [27].

$$AB\;(kg)=exp(-2.557+0.94*Ln(pi*{DBH}^{2}*H))$$ (7)

BB(kg)=0.21*AB (8)

Where AB is the aboveground biomass (which includes trunk and branches) and BB is the belowground biomass of trees in kg, DBH is the diameter, Ln is the natural logarithm, H is the total height, and pi is the wood density. With the AB data of each sample, the AB increase was calculated from Eq 2.

### Measurement of litter production (NPP _litter_)

In total 125 collectors were monitored for sampling litter fall; each one was installed 1 m above the ground in the center of each subplot. The 0.5 m^2^ (1 x 0.5 m) collectors were made with PVC pipes and plastic mesh. Litter fallen in the collectors was removed each month for one year. Logistic constraints prevented us the collection at shorter intervals, so it is expected that some litter decomposition occurred before collection. Based on published data of litter decomposition in tropical wet forests [33, 34, 35], we estimated that underestimation of litter production due to the 30-day sampling interval as compared to the daily retrieval of litter fallen in traps, would be less than 10% of total litter fall. Therefore, compared to most published values of litter production, which are based on collections every two or three weeks, our underestimation would be negligible. In each sampling, the material was placed in plastic bags and separated into leaves, branches ≤ 2 cm in diameter, reproductive material, and miscellaneous, in order to estimate the relative contribution of each fraction. In a similar way, the NPP _leaves_ was determined with the leaf fraction of litter.

### Measurement of fine root production (NPP _fine roots_)

The ingrowth core method was used to measure the NPP _fine roots_ [15, 36] with a modification consisting in the introduction of coarse metallic wires in the walls of the orifice where the root-free soil is placed, which work as guides to facilitate the subsequent extraction of the same soil cylinder initially extracted and helps to control the volume of soil sample.

The ingrowth cores were placed in the center of each 10 x 10 m sampling unit; samples were extracted from 0–10 cm and 10–20 cm- depth with an Eijkelkamp soil auger (8 cm diameter and 15 cm depth). Fine roots (FR) (with diameters ≤ 5mm) that grew inside samples were separated by hand in the field with sieves of 0.5 and 1.0 mm diameter. Afterwards, the remaining soil was placed in the holes again and roots were taken to the Laboratorio de Botánica y Ecología of the Universidad Tecnológica del Chocó. This procedure was carried out every three months for one year.

In the lab, the FR samples were washed with pressurized water to remove soil and other impurities. Samples were subsequently oven—dried at 70°C for 48 hours and weighed in an analytical precision scale (0.0001 g). With dry weights, the biomass of FR (FRB) was estimated in t ha^-1^ for each sampling period; the NPP _fine roots_ was determined as the FRB produced for one year of sampling and expressed as t ha^-1^ year^-1^.

### Soil analysis

To evaluate the soil nutrient contents in each subplot, a compound sample was taken at a depth of 0–20 cm; each sample had five subsamples that were taken from the four corners and the center of the subplot. In each plot, 25 soil samples were taken for a total of 125 samples for the whole study; the analyses were carried out at the Biogeochemistry lab of the Universidad Nacional de Colombia, Medellín, with the following techniques: Bouyoucos for textural fractions, potentiometric in water solution (1:2) for pH, Walkley and Black for OM, Micro- Kjeldahl for total N, ascorbic acid in an UV-VIS spectrophotometer after extraction with the Bray II method for available P, atomic absorption for Ca, Mg, and K extracted with ammonium acetate [37].

### Statistical analysis

The variation of NPP (total and per component) as a function of the localities was evaluated with the non-parametric Mann-Whitney (*W*) test [38]. To assess the relationship between NPP (total and per component) and soil variables (texture, OM, pH, Al, P, Ca, K, Mg, effective cation exchange capacity–ECEC-), the Spearman's rank correlation coefficient was used (*R*_*s*_) because data did not comply the assumptions of normality and homogeneity of variances evaluated with the statistics of *Bartlett*, *Hartley* and *Kurtosis* [38]. Subsequently, a principal components analysis (PCA) was used to evaluate the linear relationships between edaphic variables and the aboveground and belowground NPP. The analyses were carried out with Statgraphics Centurion XV and R [39].

## Results

### Edaphic conditions

The soils of both localities were characterized by low ECEC. Likewise, they presented low concentrations of P, Mg, and Ca; while K values were intermediate. The edaphic concentrations of P and Ca were similar in both localities; the rest of the soil variables presented significant differences (Table 2). In particular, in Pacurita, soils presented extreme acidity, high percentages of Al saturation (*57*.*2%*) and high contents of silt and clay. While in Opogodó, soils had more sand with higher concentrations of OM and total N (OM = 11.9%; N = 0.61%). Based on the lower acidity, the high contents of OM and total N, and the intermediate values of K, the soils in Opogodó were considered more fertile (Table 2).

**Table 2:** Soil characteristics in two tropical rainforests of the Colombian Pacific region.

Variables	Opogodó		Pacurita		Test
	Mean ± SE	Range	Mean ± SE	Range	
pH	4.97 ± 0.03	4.22–5.51	4.03 ± 0.02	3.68–4.37	-1869.0***
Aluminum (cmol kg^-1^)	0.12 ± 0.01	0.1–0.3	0.94 ± 0.03	0.2–1.4	1790.0***
Al Saturation (%)	12.65 ± 0.52	3.78–31.57	57.21 ± 0.91	15.6–71.06	1786.0***
Organic matter (%)	11.94 ± 0.44	4.61–24.74	4.06 ± 0.17	1.95–5.85	-1816.0***
Nitrogen (%)	0.61 ± 0.02	0.23–1.68	0.20 ± 0.01	0.1–0.29	-1815.0***
Phosphorus (ppm)	1.32 ± 0.06	0.63–3.5	1.36 ± 0.09	0.49–3.2	43.5ns
Potassium (cmol kg^-1^)	0.23 ± 0.01	0.06–0.48	0.17 ± 0.01	0.03–0.47	-796.0***
Magnesium (cmol kg^-1^)	0.28 ± 0.02	0.12–1.85	0.18 ± 0.01	0.06–0.35	-964.0***
Calcium (cmol kg^-1^)	0.38 ± 0.02	0.06–0.96	0.35 ± 0.01	0.17–0.79	-89.0ns
ECEC (cmol kg^-1^)	1.03 ± 0.04	0.56–2.64	1.64 ± 0.03	0.77–2.19	1474.5***
Clay (%)	1.04 ± 0.27	0.0–12.0	18.52 ± 0.52	10.0–28.0	1772.5***
Silt (%)	13.23 ± 0.59	4.0–28.0	28.12 ± 0.87	8.0–40.0	1626.0***
Sand (%)	85.71 ± 0.78	62.0–96.0	53.36 ± 0.95	42.0–70.0	-1763.5***
Number of samples	75		50		

Data are means ± standard error. Asterisks represent significant differences for the Mann-Whitney test.

*: *p* < 0.05

**: *p* < 0.01

***: *p* < 0.0001

ns: *p* > 0.05.

### Net primary productivity

**Fig 1:**
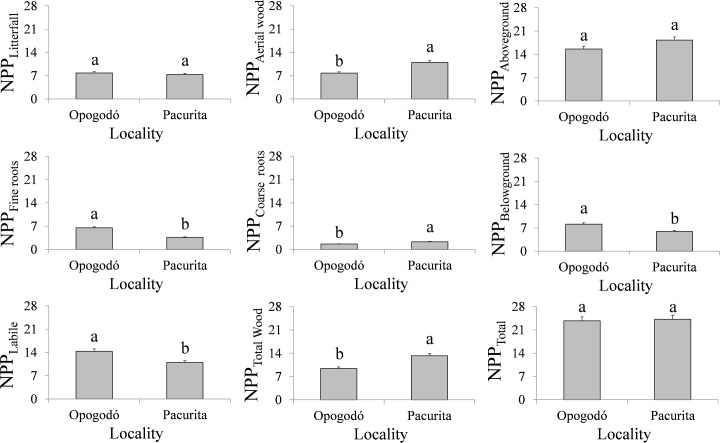
Net primary productivity (t ha^-1^ year^-1^) in two tropical rain forests in Chocó, Colombia. The gray bars are the average and vertical lines represent the standard error.

The forests of Opogodó presented a total NPP (mean *± SD*) of 23.7±2.7 t ha^-1^year^-1^; while in Pacurita it was 24.2±2.5 t ha^-1^year^-1^ with no significant differences between them (*W* = 4.0; *p* = 0.9859) (Fig 1). The NPP _wood_ in Opogodó was 7.8±2.2 t ha^-1^year^-1^; while in Pacurita, it was significantly higher (10.9±2.1 t ha^-1^year^-1^; *W* = 440.0; *p* = 0.026) (Fig 1). The NPP _litter_ was 7.8 ± 0.2 t ha^-1^year^-1^ in Opogodó, similar to that in the forests of Pacurita (*W = -193*.*0; p = 0*.*331*) with 7.4±0.2 t ha^-1^year^-1^ (Fig 1). The distribution of the components of NPP _litter_ was also similar in both localities: ≈63% leaves, 19% stems, 4% reproductive material and 13% miscellaneous (Table 3).

**Table 3:** NPP _litter_ and its components in tropical pluvial forests of Opogodó and Pacurita in Chocó, Colombia.

Opogodó	Leaves	Wood	Reproductive Material	Miscellaneous	Total
NPP _litter_ (t ha^-1^ year^-1^)	4.98	1.48	0.30	1.07	7.82
%	63.59	18.98	3.81	13.62	100.00
Standard deviation	0.99	1.00	0.21	0.64	2.05
**Pacurita**					
NPP _litter_ (t ha^-1^ year^-1^)	4.58	1.35	0.41	1.01	7.35
%	62.33	18.42	5.59	13.67	100.00
Standard deviation	1.26	0.74	0.42	0.29	1.70

The NPP _coarse roots_ was higher (*W = 440*.*0; p = 0*.*0267*) in Pacurita than in Opogodó (2.29±0.46 and 1.63±0.4 t ha^-1^year^-1^, respectively. Fig 1). On the other hand, the NPP _fine roots_ was significantly higher (*W = -1614*.*0; p = 0*.*0000*) in Opogodó than in Pacurita (6.5±0.3 t ha^-1^year^-1^ and 3.6±0.1 t ha^-1^year^-1^, respectively. Fig 1).

The NPP _aboveground_ (NPP _litter_ + NPP _wood_) in Opogodó was 15.6±2.2 t ha^-1^year^-1^, equivalent to 65.7% of total NPP in those forests; in Pacurita, the NPP _aboveground_ was 18.3±2.1 t ha^-1^year^-1^, equivalent to 75.6% of total NPP. However, the differences in the NPP _aboveground_ between zones were not significant (*W = 329*.*0; p = 0*.*0978*) (Fig 1). On the other hand, the NPP _belowground_ (NPP _fine roots_ + NPP _coarse roots_) was higher (*W = -894*.*0; p = 0*.*0000*) in Opogodó than in Pacurita (8.1±0.5 and 5.9±0.4 t ha^-1^year^-1^, respectively) (Fig 1).

The NPP _total wood_ (NPP _wood_ + NPP _coarse roots_) was higher (*W = 440*.*0; p = 0*.*0267*) in the forest of Pacurita, with 13.2±2.5 t ha^-1^year^-1^; which represented 54.7% of total NPP; in Opogodó, it was 9.4±2.6 t ha^-1^year^-1^ and represented 39.7% of total NPP (Fig 1). Finally, the NPP _labile material_ (NPP _litter_ + NPP _fine roots_) was higher (*W = -1309*.*0; p = 0*.*0000*) in Opogodó, with 14.3±0.4 t ha^-1^year^-1^ and represented 60.3% of total NPP in this location; in Pacurita, it was 10.9±0.2 t ha^-1^year^-1^ and represented 45.3% of total NPP (Fig 1).

### Relationship between NPP and soil variables

Total NPP did not present a significant correlation with the edaphic variables, as opposed to the NPP components, which had significant correlation with some of the soil variables (Table 4). In particular, the NPP _litter_ presented a significant, weak positive correlation with sand (*r = 0*.*19; p<0*.*05*) and a negative one with silt (*r = -0*.*24; p<0*.*05*). While NPP _leaves_ had a positive relationship with pH, OM, total N, Mg, and sand, and a negative association with the Al, ECEC, and silt (Table 4). The NPP _wood_ presented a weak negative correlation with pH (Table 4); NPP _coarse roots_ and NPP _total wood_ had equal correlations because the first variable was calculated from the second one. The NPP _fine roots_, NPP _belowground_, NPP _labile material_, and percentage of NPP _belowground_ had a positive association with pH, OM, total N, K, Mg, and sand, and had a negative correlation with Al, ECEC, silt, and clay (Table 4).

**Table 4:** Spearman rank correlations of components of net primary production and soil nutrients in two tropical rain forests in Chocó, Colombia.

Components of NPP	pH	Al	OM	N	P	K	Ca	Mg	ECEC	Sand	Silt	Clay
*NPP*_*litter*_	0.16ns	-0.12ns	0.09ns	0.11ns	-0.10ns	0.03ns	0.13ns	0.05ns	-0.02ns	0.19*	-0.24*	-0.03ns
*NPP*_*leaves*_	0.22*	-0.31**	0.24*	0.26**	-0.03ns	0.07ns	-0.03ns	0.19*	-0.23*	0.27**	-0.31**	-0.16ns
*NPP*_*wood*_^*1*^	-0.24**	0.15ns	-0.09ns	-0.10ns	0.05ns	0.01ns	0.01ns	-0.07ns	0.16ns	-0.13ns	0.12ns	0.16ns
*NPP*_*aboveground*_	-0.18*	0.10ns	-0.05ns	-0.04ns	0.01ns	0.04ns	0.06ns	-0.06ns	0.14ns	-0.09ns	0.07ns	0.15ns
*NPP*_*fine roots*_	0.62***	-0.68***	0.59***	0.61***	-0.11ns	0.35***	0.02ns	0.39***	-0.48***	0.62***	-0.57***	-0.66***
*NPP*_*belowground*_	0.28**	-0.38***	0.36***	0.37***	-0.07ns	0.25**	-0.01ns	0.19*	-0.24**	0.33***	-0.30**	-0.36***
*NPP*_*labile*_	0.55***	-0.58***	0.49***	0.52***	-0.11ns	0.29**	0.11ns	0.35***	-0.36***	0.54***	-0.52***	-0.51***
*NPP*_*total*_	-0.06ns	-0.03ns	0.05ns	0.06ns	-0.01ns	0.12ns	0.03ns	0.01ns	0.04ns	0.02ns	-0.02ns	0.01ns
%*NPP*_*belowground*_	0.51***	-0.47***	0.39***	0.41***	-0.09ns	0.19*	-0.03ns	0.25**	-0.37***	0.43***	-0.38***	-0.49***

^*1*^ Correlations for *NPP*_*coarse roots*_ and *NPP*_*total Wood*_ are identical to those for *NPP*_*wood*_ as much as the first two variables were estimated from the last one.

Asterisks and bold letters represent significant correlations.

*: *p* < 0.05

**: *p* < 0.01

***: *p* < 0.0001

ns: *p* > 0.05

**Fig 2:**
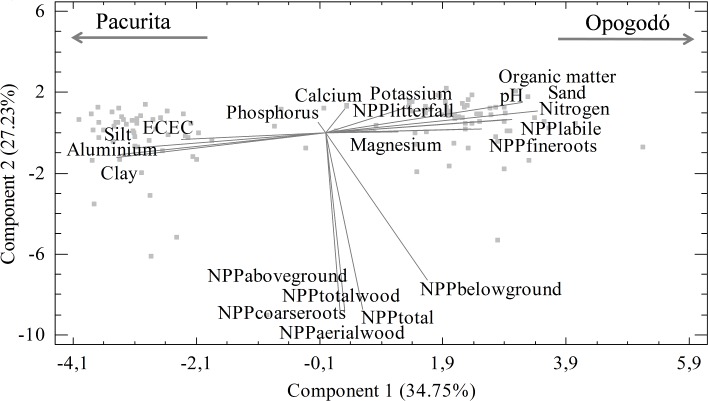
Principal component analysis of edaphic variables and components of net primary productivity in two pluvial tropical forests of Chocó, Colombia.

The PCA showed that the evaluated variables formed a gradient of edaphic conditions in the first component, with a high content of Al, clay, silt, and ECEC in the soils of Pacurita; on the other hand, contents of sand, pH, OM and total N were higher in Opogodó (Fig 2). Likewise, the vectors of NPP _fine roots_ and NPP _labile material_ presented an analogous tendency to the soils of Opogodó, which suggests a possible association between these variables. However, the other variables of the NPP mainly aligned with the second principal component, suggesting a slight association with the edaphic variables evaluated. The first two components explained 59.4% of the total variability; although the first six components had eigenvalues higher than 1, each of the last 4 components explained less than 10% of the variance and were not included in the analysis.

## Discussion

### Does this study provide evidence that high precipitation decreases the NPP in tropical forests?

The total NPP values recorded in the pluvial forests of Chocó (23.7 t ha^-1^year^-1^ in Opogodó and 24.2 t ha^-1^year^-1^ in Pacurita) were within the range of 8.4–33.0 t ha^-1^year^-1^ reported for tropical forests with less precipitation and within the confidence interval of 19.84–25.9 t ha^-1^year^-1^ of total NPP for tropical forests with annual precipitation between 707 and 3,565 mm [40]. The results suggest that NPP of the forests of Chocó is similar to that reported in other tropical forests with less precipitation. Therefore, they do not support the hypothesis of lower NPP of tropical forests with precipitation greater than 5,000 mm per year [7].

Different processes could explain the similarity of NPP between the forests of Chocó and other tropical forests with lower rainfall: first, it is expected that the reduction of O_2_ in the soil by intense rainfall [7, 41] could result in plant stress by root anoxia and O_2_ limitation for the decomposition of OM with a negative effect on NPP. This situation could be particularly important in plots of Opogodó, where topography is flat (Table 1); however, steep topography such as that of soils of Pacurita facilitates drainage and run-off, which would dampen the O_2_ limitation. Even in the flat plots of Opogodó, water-logging conditions depend on the temporal distribution of rainfall, characterized by two peaks of high rainfall and two periods of low precipitation [20]; the sandy texture of those plots (Table 2) facilitates the fast drainage of soils. Similar NPP values among flood plain forest types with different inundation periods (1, 2, and 4 months per year) reported in Peruvian amazon [42], support this argument.

Second, given that high precipitation results in excessive losses of soil nutrients by leaching and run-off [17, 19], to counterbalance this limitation, pluvial forests of Chocó should have developed a higher productivity, growth, and longevity (lifetime) of FR [43]. This phenomenon was more evident in the sandy soils of Opogodó, where the higher rates of NPP _fine roots_ contributed to compensate the lower NPP _wood_, which resulted in similar values of total NPP between sites. The influence of edaphic conditions on NPP _belowground_ is discussed in more detail below.

Third, the NPP depends on the absorbed photosynthetically active radiation [44]; therefore, it is expected that the cloudiness typical of rainy areas decreases the incident radiation. However, the precipitation levels in the pluvial forests of Chocó vary significantly throughout the day, so that there are more frequent and intense rains at nighttime [45]. Consequently, in the hours of more sunlight, between 10:00 am and 3:00 pm, the photosynthesis and, therefore, the NPP would not be inhibited by the excessive rainfall of the region.

### To what extent do the edaphic conditions explain the NPP of tropical forests with high precipitation?

In the present study, the total NPP did not present a significant association with any of the edaphic variables evaluated; therefore, the hypothesis that the availability of soil P limits the total NPP of lowland tropical rainforests was not confirmed in the plots evaluated here [9, 10, 11, 14]. This low association was probably due to the lack of a true gradient of available edaphic P between the study areas; in both localities, P concentrations were similar and very low (range of 0.49–3.5 ppm) (Table 2). In order to evaluate the nutritional limitation of NPP in these areas, it would be necessary to monitor it after the addition of nutrients to the soil, similar to other few works done in lowland tropical forests [46, 47].

The high diversity of tree species in the forests of this region, with 100–300 species recorded per hectare, could also partly explain the slight relationship observed between total NPP and soil variables [48, 49]; as a consequence, heterogeneous growth responses would have occurred as a consequence of variations in nutrient availability (heterogeneous nutrient limitation) [47], which are determined by species, age, and functional group.

Despite the time scale of the sampling of this study is one year, which seems too short to be representative of the long-term relationship of NPP and its components with soil nutrients, the number of samples is large (125 distributed in 5 1-ha permanent plots), which are representative of the variation of soil conditions in the study area. Therefore, although components of NPP might vary among years, there is no reason to expect that the relations found here change significantly from one year to another.

### To what extent do the edaphic conditions in tropical forests with high precipitation influence the amount of NPP _belowground_?

Despite total NPP did not show a significant relationship with soil, the aboveground and belowground components of NPP presented a significant association with edaphic variables (Table 4). The components related to NPP _fine roots_ (NPP _belowground_, NPP _labile material_ and %NPP _belowground_) were more significantly associated with soil variables (Al, pH, OM, total N, K, Mg, ECEC, sand, silt, and clay), which evidenced that this is the component of NPP more sensitive to changes in the edaphic conditions. Similarly, in forests of the Amazon, it has been reported that the relationship between NPP and edaphic conditions varied in accordance with the component evaluated and that fine roots were the most sensitive, mainly to the availability of edaphic P [14].

The increase in the NPP _fine roots_ with the availability of nutrients (total N, K, and Mg) has been previously reported [50]; these authors demonstrated that the NPP of this component increases with the edaphic content of nutrients such as N, P, Ca and Mg in Amazonian forests. Likewise, it has been reported a higher growth of FR in soils with high rates of supply of the ions NH_4_^+^, PO_4_^3-^ and K^+^ in forests of Malaysia [43]. These trends in the relationship of NPP _fine roots_ with nutrients in tropical oligotrophic soils are probably due to the fact that in conditions of low soil fertility, FR tend to rapidly expand into small patches of nutrient- rich soil [43, 51], which would reduce the nutritional deficit.

The increase in the NPP _leaves_ and NPP _fine roots_ with the local availability of several nutrients (total N, K, and Mg) suggests that NPP is limited by multiple nutrients (not only by edaphic P), which is similar to other reports [46, 47, 52, 53, 54]. In particular, the limitation of NPP by total N in the studied forests could obey to a low fixing rate of atmospheric N in the ecosystem caused by the low availability of edaphic P and other mineral nutrients [55], which should be the subject of future studies. Under these limiting nutrient conditions, the NPP _leaves_ and NPP _fine roots_ tended to increase along with the increase in the local contents of sand and OM.

NPP _belowground_ did not decrease with the increase in the local availability of total N and OM (Fig 2) as indicated by the differential allocation hypothesis [15], but rather increased. This result suggests that with the increase in soil fertility at local scale, the higher NPP of root components is a mechanism for capturing more nutrients in oligotrophic conditions. As a consequence, these results seem to support the “constant allocation hypothesis” at local scale, which states that the high edaphic fertility increases the total NPP, but the amount of the NPP _aboveground_ and NPP _belowground_ remains relatively constant [15].

On the other hand, at the landscape scale, the forests with different textured soils of this study showed significant differences in the components of the NPP, with higher NPP _belowground_ in the more sandy soils of Opogodó and higher NPP _total wood_ in Pacurita (Fig 1). Likewise, important edaphic controls on carbon allocation of two Amazon forests under similar climatic conditions on contrasting soils (clayey versus sandy soils) has been reported [56]. In particular, they found that NPP, fine litterfall and the increment of aboveground biomass were higher in the clayey soil forest; while, fine-root production was higher in the white-sand forest.

In summary, this study supports the hypothesis that at the landscape scale, soils control a trade-off between carbon allocation to fine roots *versus* aboveground biomass increment [56], which could explain the similitude of NPP among sites with different environmental conditions; its detailed examination is an important area of research in order to deepen our understanding of factors governing NPP across the globe.

### Why were there differences in the NPP _total wood_ but not in the NPP _litter_ between the localities of Opogodó and Pacurita?

In Opogodó topography was flatter (Table 1) and there were some pooling of water and ravines close to the plots; therefore, the intense rainfall produces puddles, swamps and probably temporal anoxia conditions in that locality. Reduction of soil O_2_ caused by the combination of high rainfall and flat topography has been observed elsewhere [41] even several days after the rainfall. Under flooding and water pooling conditions on soils, the O_2_ availability decreases rapidly, which alters the metabolism of plants and inhibits their growth as a consequence of stomatal closure, reduction of photosynthesis, translocation of carbohydrates and absorption of nutrients from the soil [57, 58]. These circumstances reduce the elongation of internodes and induce chlorosis, causing premature senescence and foliar abscission [58], and therefore cambial dormancy and reduction of tree growth [59]. Consequently, the seasonal conditions of stress from anoxia in Opogodó possibly affected the growth of tree stems and reduced the NPP _total wood_.

On the other hand, NPP litter was similar between localities, which agrees with other studies where litterfall was not different between forests on different soil types. For example, in a meta-analysis of litterfall patterns in tropical South America [60], litterfall was not different between old- growth tropical rainforests (8.61±1.91 Mg ha^−1^ yr^−1^) and flooded tropical forests (8.89±1.42 Mg ha^−1^ yr^−1^). Litterfall was also not different among three flood plain forest types with different inundation periods (1, 2 and 4 months per year) in Peruvian amazon [42]. Litterfall has even been higher in seasonally flooded than in non-flooded tropical forests of Pantanal (Brazil) [61]. These results suggest that seasonal water excess is not limiting for litter production; on the contrary, flooding conditions promote leaf abscission [58]. However, cambial dormancy resulting from the stress-inducing anoxic conditions seem to decrease NPP _wood_. Therefore, under such conditions, carbon allocation seem to change from woody tissue to labile tissue (leaves and fine roots), which makes sense as much as the long term survival depends on the capacity for nutrient absorption and photosynthesis.

## Supporting Information

S1 Dataset Data of NPP components and soil variables per subplot. (XLSX)

## References

[1] Saugier B, Roy J, Mooney HA. Estimations of global terrestrial productivity: converging toward a single number? In: Roy J, Saugier B, Mooney HA, editors. Terrestrial Global Productivity. San Diego: Academic Press; 2001 pp. 543–557.

[2] Pan Y, Birdsey RA, Fang J, Houghton R, Kauppi PE, Kurz WA, et al A large and persistent carbon sink in the world’s forests. Science 2011; 333: 988–993. 10.1126/science.1201609 21764754

[3] Field CB, Behrenfeld MJ, Randerson JT, Falkowski P. Primary production of the biosphere: integrating terrestrial and oceanic components. Science 1998; 281: 237–240. 9657713 10.1126/science.281.5374.237

[4] Phillips OL, Malhi Y, Higuchi N, Laurance W, Núñez P, Vásquez M, et al Changes in the carbon balance of tropical forests: evidence from long-term plots. Science 1998; 282: 439–442. 9774263 10.1126/science.282.5388.439

[5] Clark DA, Brown S, Kicklighter DW, Chambers JD, Thomlinson JR, Ni J. Measuring net primary production in forests: concepts and field methods. Ecol Appl 2001; 11: 356–370.

[6] Clark DA, Brown S, Kicklighter DW, Chambers JD, Thomlinson JR, Ni J, et al Net primary production in forests: an evaluation and synthesis of existing field data. Ecol Appl 2001; 11: 371–384.

[7] Schuur EAG. Net primary productivity and global climate revisited: the sensitivity of tropical forest growth to precipitation. Ecology 2003; .84: 1165–1170.

[8] Zhao M, Running SW. Drought-induced reduction in global terrestrial net primary production from 2000–2009. Science 2010; 329: 940–943. 10.1126/science.1192666 20724633

[9] Cleveland CC, Townsend A, Taylor P, Alvarez-Clare S, Bustamante M, Chuyong G, et al Relationships among net primary productivity, nutrients and climate in tropical rain forest: a pan-tropical analysis. Ecol Lett 2011; 14: 939–947. 10.1111/j.1461-0248.2011.01658.x 21749602

[10] Vitousek PM. Litterfall, nutrient cycling, and nutrient limitation in tropical forests. Ecology 1984; 65: 285–298.

[11] Vitousek PM, Sanford RL. Nutrient cycling in moist tropical forest. Annu Rev Ecol Syst 1986; 17: 137–167.

[12] Chapin FS III, Vitousek PM, Van Cleve K. The nature of nutrient limitation in plant communities. Am Nat 1986; 127: 48–58.

[13] Paoli GD, Curran LM, Zak DR. Phosphorus efficiency of aboveground productivity in Bornean rain forest: evidence against the unimodal efficiency hypothesis. Ecology 2005; 86: 1548–1561.

[14] Aragão LEO, Malhi Y, Metcalfe DB, Silva-Espejo JE, Jiménez E, Navarrete D, et al Above- and below-ground net primary productivity across ten Amazonian forests on contrasting soils. Biogeosciences 2009; 6: 2441–2488.

[15] Hendricks JJ, Hendrick RL, Wilson CA, Mitchell RJ, Pecot SD, Guo D. Assessing the patterns and controls of fine root dynamics: an empirical test and methodological review. J Ecol 2006; 94: 40–57.

[16] Alvarez-Clare S, Mack MC. Influence of precipitation on soil and foliar nutrients across nine Costa Rican forests. Biotropica 2011; 43: 433–441.

[17] Austin AT, Vitousek PM. Nutrient dynamics on a precipitation gradient in Hawaii. Oecologia 1998; 113: 519–529. 28308032 10.1007/s004420050405

[18] Buol SW, Hole FD, McCracken RJ. Génesis y Clasificación de Suelos. México DF: Editorial Trillas; 1981.

[19] Posada JM, Schuur EAG. Relationships among precipitation regime, nutrient availability, and carbon turnover in tropical rain forests. Oecologia 2011; 165:783–795. 10.1007/s00442-010-1881-0 21207233

[20] Poveda IC, Rojas C, Rudas A, Rangel O. 2004 El Chocó biogeográfico: ambiente físico In: Rangel O, editor. Colombia Diversidad Biótica IV. El Chocó biogeográfico/ Costa Pacífica. Bogotá: Instituto de Ciencias Naturales, Universidad Nacional de Colombia; 2004. pp. 1–22.

[21] West R. Las tierras bajas del Pacífico colombiano. Bogotá: Instituto Colombiano de Antropología; 1957.

[22] Martínez JO. Geomorfología In: Leyva P, editor. Colombia Pacífico, Tomo I. Santafé de Bogotá: Fondo para la Protección del Medio Ambiente “José Celestino Mutis” FEN Colombia; 1993.

[23] Instituto Geográfico Agustín Codazzi. Mapa de Suelos de Colombia. Bogotá: IGAC; 2002.

[24] Ruiz–Murcia J. Cambio Climático en temperatura, precipitación y humedad relativa para Colombia usando modelos meteorológicos de alta resolución (Panorama 2011–2100). Bogotá: Instituto de Hidrología, Meteorología y Estudios Ambientales–IDEAM; 2010.

[25] Gardi C, Angelini M, Barceló S, Comerma J, Cruz Gaistardo C, Encina Rojas A, et al Atlas de suelos de América Latina y el Caribe. Luxembourg: Comisión Europea -Oficina de Publicaciones de la Unión Europea; 2014.

[26] Sierra CA, Harmon ME, Moreno FH, Orrego SA, del Valle JI. Spatial and temporal variability of net ecosystem production in a tropical forest: testing the hypothesis of a significant carbon sink. Glob Change Biol 2007; 13: 838–853.

[27] Quinto-Mosquera H, Moreno-Hurtado F. Dinámica de la biomasa aérea en un bosque pluvial tropical del Chocó Biogeográfico. Rev Facultad Nacional de Agronomía Medellín 2011; 64: 5917–5936.

[28] Gentry AH. A field guide to the families and genera of woody plants of Northwest South America (Colombia, Ecuador, Peru), with supplementary notes on herbaceous taxa. Washington: Conservation International; 1993.

[29] Baker TR, Phillips OL, Malhi Y, Almeida S, Arroyo L, Di Fiore A, et al Variation in wood density determines spatial patterns in Amazonian forest biomass. Glob Change Biol 2004; 10: 545–562.

[30] Brown S. Estimating biomass and biomass change of tropical forests: A Primer. Roma: Food and Agriculture Organization (UN FAO Forestry Paper; no. 134); 1997.

[31] Chambers JQ, Dos Santos J, Ribeiro RJ, Higuchi N. Tree damage, allometric relationships, and above-ground net primary production in central Amazon forest. Forest Ecol Manag 2001; 152: 73–84.

[32] Chave J, Andalo C, Brown S, Cairns MA, Chambers JQ, Eamus D, et al Tree allometry and improved estimation of carbon stocks and balance in tropical forests. Oecologia 2005; 145: 87–99. 10.1007/s00442-005-0100-x 15971085

[33] Aerts R. Climate, leaf litter chemistry and leaf litter decomposition in terrestrial ecosystems: a triangular relationship. Oikos 1997; 79:439–449.

[34] Sundarapandian SM, Swamy PS. Litter production and leaf-litter decomposition of selected tree species in tropical forests at Kodayar in the Western Ghats, India. Forest Ecol Manag 1999; 123: 231–244.

[35] Wieder W, Cleveland C, Townsend A. Controls over leaf litter decomposition in wet tropical forests. Ecology 2009; 90:3333–41. 20120803 10.1890/08-2294.1

[36] Cuevas E, Medina E. Nutrient dynamics within Amazonian forests. II. Fine root growth, nutrient availability and leaf litter decomposition. Oecologia 1988; 76: 222–235. 28312200 10.1007/BF00379956

[37] Osorio NW. Manejo de nutrientes en suelos del Trópico. 2nd ed. Medellín: Editorial L. Vieco SAS; 2014.

[38] Hoshmand AR. Statistical Methods for Environmental & Agricultural Sciences. 2nd ed. New York: CRC Press LLC; 1998.

[39] The R Project for Statistical Computing. R: a language and environment for statistical computing. Vienna, Austria. Available: http://www.r-project.org/. Accessed 20 September 2015.

[40] Malhi Y, Doughty C, Galbraith D. The allocation of ecosystem net primary productivity in tropical forests. Philos T Roy Soc B 2011; 366: 3225–3245. 10.1098/rstb.2011.0062 PMC3179639 22006964

[41] Silver W, Lugo A, Keller M. Soil oxygen availability and biogeochemistry along rainfall and topographic gradients in upland wet tropical forest soils. Biogeochemistry 1999; 44: 301–328.

[42] Nebel G, Dragsted J, Vega AS. Litte1r fall, biomass and net primary production in flood plain forests in the Peruvian Amazon. Forest Ecol Manag 2001; 150: 93–102.

[43] Kochsiek A, Tan S, Russo SE. Fine root dynamics in relation to nutrients in oligotrophic Bornean rain forest soils. Plant Ecol 2013; 214: 869–882.

[44] Landsberg JJ, Prince SD, Jarvis PG, McMurtrie RE, Luxmoore R, Medlyn BE. Energy conversion and use in forests: an analysis of forest production in terms of radiation utilization efficiency In: Gholz HL, Nakane K, Shimoda H, editors. The Use of Remote Sensing in the Modeling of Forest Productivity. Dordrecht: Kluwer Academic; 1996 pp. 273–298.

[45] Murillo W, Palomino R, Córdoba S, Aragón G, Banguero E. El régimen diario de la precipitación en el municipio de Quibdó (Colombia). Rev de Climatología 2005; 5: 1–7.

[46] Mirmanto E, Proctor J, Green J, Nagy L, Suriantata. Effects of nitrogen and phosphorus fertilization in a lowland evergreen rainforest. Philos T Roy Soc B 1999; 354:1825–1829. 10.1098/rstb.1999.0524 PMC1692691 11605625

[47] Alvarez-Clare S, Mack MC, Brooks M. A direct test of nitrogen and phosphorus limitation to net primary productivity in a lowland tropical wet forest. Ecology 2013; 94: 1540–1551. 23951714 10.1890/12-2128.1

[48] Faber-Langendoen D, Gentry AH. The structure and diversity of rain forests at Bajo Calima, Chocó region, western Colombia. Biotropica 1991; 23: 2–11.

[49] Quinto-Mosquera H, Moreno-Hurtado F. Diversidad florística arbórea y su relación con el suelo en un bosque pluvial tropical del Chocó biogeográfico. Rev Árvore 2014; 38: 1123–1132.

[50] Metcalfe D B, Meir P, Aragao LE, Da Costa ACL, Braga AP, Gonḉalves PHL, et al The effects of water availability on root growth and morphology in an Amazon rainforest. Plant Soil 2008; 311: 189–199.

[51] Robinson D, Hodge A, Griffiths BS, Fitter AH. Plant root proliferation in nitrogen-rich patches confers competitive advantage. P Roy Soc Lond B Bio 1999; 266: 431–435.

[52] Paoli GD, Curran LM. Soil nutrients limit fine litter production and tree growth in mature lowland forest of southwestern Borneo. Ecosystems 2007; 10: 503–518.

[53] Kaspari M, Garcia MN, Harms KE, Santana M, Wright SJ, Yavitt JB. Multiple nutrients limit litterfall and decomposition in a tropical forest. Ecol Lett 2008; 11: 35–43. 10.1111/j.1461-0248.2007.01124.x 18021246

[54] Wright SJ, Yavitt JB, Wurzburger N, Turner BL, Tanner EVJ, Sayer EJ, et al Potassium, phosphorus, or nitrogen limit root allocation, tree growth, or litter production in a lowland tropical forest. Ecology 2011; 92: 1616–1625. 21905428 10.1890/10-1558.1

[55] Vitousek P, Porder S, Houlton B, Chadwick O. Terrestrial phosphorus limitation: mechanisms, implications, and nitrogen–phosphorus interactions. Ecol Appl 2010; 20: 5–15. 20349827 10.1890/08-0127.1

[56] Jiménez EM, Peñuela MC, Sierra CA, Lloyd J, Phillips OL, Moreno FH, et al Edaphic controls on ecosystem-level carbon allocation in two contrasting Amazon forests. J. Geophys. Res. Biogeosci 2014; 119: 1–11.

[57] Kozlowski T. Plant responses to flooding of soil. BioSciences 1984; 34: 162–167.

[58] Kozlowski T. Responses of woody plants to flooding and salinity. Tree Physiol Monograph 1997; 1: 1–29.

[59] Kozlowski T, Pallardy SG. Growth control in woody plants. San Diego: Academic Press; 1997.

[60] Chave J, Navarrete D, Almeida S., Álvarez E, Aragão LEOC, Bonal D, et al Regional and seasonal patterns of litterfall in tropical South America. Biogeosciences 2010; 7: 43–55.

[61] Haase R. Litterfall and nutrient return in seasonally flooded and non- flooded forest of the Pantanal, Mato Grosso, Brazil. Forest Ecol Manag 1999; 117: 129–147.

