# Correction: Net Primary Productivity and Edaphic Fertility in Two Pluvial Tropical Forests in the Chocó Biogeographical Region of Colombia

**DOI:** 10.1371/journal.pone.0175620

**Published:** 2017-04-06

**Authors:** Harley Quinto-Mosquera, Flavio Moreno

S1 Dataset appears incorrectly. Please view the correct S1 Dataset below.

## Supporting information

S1 Dataset Data of NPP components and soil variables per subplot. (XLSX)
